# Intelligence

**Published:** 1809-07

**Authors:** 


### INTELLIGENCE.

Mr. Joseph Hume has discovered a new method of detecting arsenic. The test which he proposes as a substitute for those hitherto used, appears to be more efficacious, inasmuch as it produces a more copious precipitate from a given quantity of that substance. It is composed in the following manner: Let one grain of white oxide of arsenic, and the same quantity of carbonate of soda, be dissolved by boiling in ten or twelve ounces of distilled water, which ought to be done in a glass vessel; to this let a small quantity of the nitrate of silver be added, and a bright yellow precipitate will instantly appear.

M. Vauquelin has examined the root of a species of polypody, known by the appellation of calaguala. Of the substances which compose it, only those soluble in alcohol and water are capable of producing any effect on the animal economy. These are saccharine matter, mucilage, muriate of potash, and resin, which last he conjectures would be found to destroy the tape worm. He has likewise made similar experiments on the roots of the common polypody and male fern, and obtained from them precisely similar principles and nearly in the same proportions as from the calaguala. The former roots, however, contain a small quantity of tannin. Thus the medicinal virtues of the calaguala root being analagous to those of the other ferns, is fully confirmed by chemical analysis.

The Journal de Medicine contains several cases of coincidence in Variola and Vaccina. These are related with much accuracy, and exactly confirm what has been long since remarked by Doctors Woodville and Adams. The experiment of inoculating sheep has been repeated, and is said to have secured them from the scab.

The same Journal contains very high recommendations of the Radix Rhattaniæ, from observations made by M. Pager and others. We shall give the substance of them in our next.

The College of Physicians have, on the recommendation of Sir Lucas Pepys, their President, admitted a Physician to the town practice, without passing through the academic forms required by their Statutes. Dr. Adams, the author of "Morbid Poisons," &c. is the person they have thought proper to distinguish.

Dr. Clough will commence his next Course of Lectures on Puerperal Anatomy, Physiology, and Pathology, on Monday the 7th of August, at ten in the morning, at his house, No. 68, Berners Street; and for the convenience of such Students as may be engaged in attending other Classes, his Evening Course will be continued as usual on Tuesdays, Thursdays, and Saturdays, at eight. These Lectures will be elucidated by appropriate specimens of anatomical preparations and casts from Nature, from the collection of a very celebrated Teacher in this country. Particulars may be had as above, and at the St. Mary-le-bone Dispensary, Welbeck-street, Cavendish-square.

Dr. SQUIRE, Ely Place, Holborn, will begin a Course of Lectures on the Theory and Practice of Midwifery, and the Diseases of Women and Children, on Tuesday, July the 11th.

Dr. ROBERTON has in the press a new work, which will soon be published, entitled, A practical Treatise on the Anatomy, Physiology, Pathology, and practical Treatment of the Diseases of the Generative System, particularly Lues Venerea, Gonorrhœa, Gleet, Seminal Emissions, Stricture in the Urethra of every Species; also Leucorrhœa, and various other Female Complaints.

